# Patients’ IgLON5 autoantibodies interfere with IgLON5-protein interactions

**DOI:** 10.3389/fimmu.2023.1151574

**Published:** 2023-03-21

**Authors:** Jon Landa, Ana Beatriz Serafim, Carles Gaig, Albert Saiz, Inga Koneczny, Romana Hoftberger, Joan Santamaria, Josep Dalmau, Francesc Graus, Lidia Sabater

**Affiliations:** ^1^ Neuroimmunology Program, Fundació de Recerca Clínic Barcelona-Institut d’Investigacions Biomèdiques August Pi i Sunyer, Universitat de Barcelona, Barcelona, Spain; ^2^ Service of Neurology, Hospital Clinic de Barcelona, Barcelona, Spain; ^3^ Division of Neuropathology and Neurochemistry, Department of Neurology, Medical University of Vienna, Vienna, Austria; ^4^ Comprehensive Center for Clinical Neurosciences and Mental Health, Medical University of Vienna, Vienna, Austria; ^5^ Department of Neurology, University of Pennsylvania, Philadelphia, PA, United States; ^6^ Institució Catalana de Recerca i Estudis Avançats (ICREA), Barcelona, Spain

**Keywords:** anti-IgLON5 antibody encephalopathy, anti-IgLON5 disease, IgG4, interactome, neurons, IgLON5 antibodies

## Abstract

**Background:**

Anti-IgLON5 disease is a rare neurological disorder characterized by autoantibodies against IgLON5, and pathological evidence of neurodegeneration. IgLON5 is a cell adhesion molecule but its physiological function is unknown. Our aim was to investigate the IgLON5 interactome and to determine if IgLON5 antibodies (IgLON5-abs) affect these protein interactions.

**Methods:**

IgLON5 interactome was investigated by mass spectrometry sequencing of proteins immunoprecipitated by IgLON5 autoantibodies using cultures of rat cerebellar granular neurons (CGNs). Shedding of IgLON5 was explored using HEK cells transfected with human IgLON5 plasmid and in CGNs. Interactions of IgLON5 with identified binding partners and IgLON5-abs effects were confirmed by immunofluorescence in transfected HEK cells and rat hippocampal neurons.

**Results:**

Patients’ IgLON5 antibodies co-precipitated all members of the IgLON family and three 3 additional surface proteins. IgLON5 predominantly establishes homomeric and heteromeric *cis* (within the cell) and *trans* (between cells)-interactions with other IgLON family members and undergoes spontaneous ectodomain shedding. Antibodies from patients with anti-IgLON5 disease prevent trans-interactions in hippocampal neurons independently of the IgLON5 IgG subclass distribution.

**Conclusions:**

We show a potentially novel pathogenic mechanism of IgLON5-abs that consists in blocking IgLON5 interactions with its binding partners. These findings extend our knowledge about the physiological role of IgLON5 and pave the way to future understanding of the pathological mechanisms of anti-IgLON5 disease.

## 1 Introduction

Autoantibodies against IgLON5 (IgLON5-abs) are the hallmark of anti-IgLON5 disease, a recently identified clinical entity in which neurodegeneration and autoimmunity converge (1). Unlike other neurological disorders targeting neuronal cell surface antigens, the clinical progression of anti-IgLON5 disease is usually chronic with limited response to immunotherapy. Furthermore, neuropathological studies in a few patients show neuronal accumulation of hyperphosphorylated tau mainly involving the brainstem and hypothalamus supporting an underlying neurodegenerative process (2).

IgLON5 is the fifth member of the IgLON family that belongs to the immunoglobulin superfamily (IgSF) of cell adhesion molecules (CAMs). IgLONs contain 3-immunoglobulin-like (Ig-like) domains attached to the membrane by a glycosylphosphatidylinositol (GPI)-anchor protein without a transmembrane domain. Shedding of the ectodomain (soluble extracellular part of the protein) of GPI-anchored proteins has been described as an important post-translational mechanism involved in adhesion and cell signaling and has been shown to occur with other IgLON family members (3, 4).

IgLON5-abs belong to the IgG1 and IgG4 subclasses (rarely IgG2). The IgG1 subclass of IgLON5-abs cause an irreversible decrease of IgLON5 clusters on the neuronal surface by crosslinking and internalization, and after long-term exposure to total purified IgG *in vitro*, the architecture of the neuronal cytoskeleton is impaired (5, 6). Although IgG4 is the predominant subclass of IgLON5-abs, the role this subclass plays in the disease remains unclear (5), but in other diseases, the main pathogenic mechanism described for IgG4 antibodies is interference with protein-protein interactions (7). Thus, the aims of our study were to investigate if IgLON5 is shed from the cell surface in normal conditions, to analyze the interactome of IgLON5, and to explore the effects of IgLON5-abs on the interaction of IgLON5 with its binding partners.

## 2 Materials and methods

### 2.1 Patients’ samples and IgG purification

Sera from two patients with anti-IgLON5 disease and 3 healthy blood donors were used for the studies of immunoprecipitation. Paired serum/CSF samples from another 4 patients with anti-IgLON5 disease, a patient with anti-NMDAR encephalitis and a patient with Alzheimer disease were used for the studies of interaction of IgLON5 with other cell surface proteins. Total IgG was purified from these 4 patients as previously described (6). The percentage of IgLON5-IgG subclass was measured as previously reported by flow cytometry (5).

### 2.2 Primary cell cultures

Primary cell cultures of rat hippocampal neurons were prepared from embryonic E18 day as previously reported (8). Briefly, dissected hippocampi were trypsinized with 0.25% trypsin (Thermo-Fisher Scientific, Waltham Massachusetts, USA) for 15 min at 37°C and mechanically dissociated. Single cell suspension was plated in P35 plates (100.000 cells) on coverslips coated with Poly-L-lysine and maintained in Neurobasal medium plus B27 (Thermo-Fisher Scientific).

Cerebellar granule neurons (CGN) were prepared from postnatal P8 Wistar rat pups following the same protocol as for hippocampal neurons but using a different plating media (Neurobasal-A plus B27 and KCl 25 mM). Cells were plated in P100 dishes at a density of 1,3x10^6^ cells/ml. Ara-C was added (10µm) 24 hours post-plating to arrest the glial cell growth.

### 2.3 Analysis of IgLON5 ectodomain shedding

HEK293 cells (HEK) were transfected with untagged human-IgLON5 plasmid (HEK-IgLON5; SC317071, Origene, Rockville, MA, USA) as reported (1) and 24 hours post-transfection, the culture medium was replaced by Opti-MEM medium (Thermo-Fisher). As a positive control of shedding, phosphoinositide-specific phospholipase C (PI-PLC) (1U/mL), which removes GPI-anchored proteins from the cell surface, was added to one HEK-IgLON5 plate. After 24 hours, the medium of HEK-IgLON5 cultures with and without PI-PLC was collected and concentrated 10-fold using Amicon Ultracentrifugal filters 30K (UFC903024, Millipore; Burlington, MA, USA). HEK-IgLON5 cells from P60 plates were lysed with cell lysis buffer (FNN0011, Thermo-Fisher) and membrane proteins were isolated with a commercial kit (A44390, Thermo-Fisher). Proteins from total cell lysate, membrane protein preparation, and culture medium were separated electrophoretically and transferred to a nitrocellulose membrane that was sequentially incubated with IgLON5 (ab122763, Abcam, Cambridge, UK, 1:500) overnight at 4°C and goat anti-rabbit HRP secondary antibody (1/1000 diluted) provided with a chemiluminescence kit (RPN2108, GE Healthcare, Chicago, IL, USA) and visualized in an ImageQuant LAS 4000 imager (GE Healthcare). The concentration of soluble IgLON5 was quantified by immunoblot comparing the signal with that produced by IgLON5 human recombinant protein Fc-tagged (rIgLON5-Fc; 9728-IG-050, R&D Systems, Minneapolis, MN, USA) of known concentration (data not shown). Similar experiments were done using cultures of CGN.

### 2.4 Identification of IgLON5 interactome using immunoprecipitation

Live CGNs were incubated with serum (dilution 1:100) containing IgLON5-abs or from healthy donors, for 1 hour at 37°C. After extensive washing, neurons were lysed and protein complexes were incubated overnight with protein A/G beads (20424, Thermo-Fischer Scientific) at 4°C. After on-beads digestion with trypsin, the beads were centrifuged and the supernatant was analyzed by mass spectroscopy at the Proteomic facility of Centre for Genomics Regulation (CRG, Barcelona) as previously described (9). *Bona-fide* IgLON5 interactors were defined as proteins pulled-down by IgLON5-abs in two or more independent immunoprecipitations and in none of the negative controls.

### 2.5 Binding of IgLON5 with interactors in HEK cells and neurons

To validate *cis*-interactions (within the same cell) of IgLON5 with identified interactors, HEK cells were co-transfected in pairs: *IGLON5* (SC317071, Origene) plus: 1) *IGLON1* (RG213594, Origene), 2) *IGLON2* (RG226879, Origene), 3) *IGLON3* (RG207618, Origene), 4) *IGLON4* (RG216034 Origene), 5) *KIDINS220* (RC216441Origene), 6) *IGSF21* (RG203559, Origene), 7) *CACNA2D2* (RC219452, Origene) and 8) *CASPR2* (SC115167, Origene), as a non-binding control. Twenty hours post-transfection, immunoprecipitations were performed as indicated above with IgLON5-abs and pulled-down proteins were electrophoresed and transferred to a nitrocellulose membrane. The presence of the interacting proteins was detected by incubating the membrane with the following antibodies (dilution 1:500, overnight at 4°C): IgLON1 (STJ94590), IgLON2 (STJ94442), IgLON3 (STJ116461), IgLON4 (STJ94394, St. John’s Laboratory, London, UK), KIDINS220 (21856-1-AP, Proteintech, Rosemont, IL, USA), IGSF21 (21465-1-AP; Proteintech), CACNA2D2 (SAB1401461, SIGMA) and CASPR2 (ab33994, Abcam) followed by incubation with appropriate secondary antibodies (dilution 1:1000) for 1 hour at room temperature. Results were developed by chemiluminescence as described above.

To investigate interactions in *trans* (between cells), non-transfected hippocampal neurons or HEK cells transfected with: IgLON1 to 5, KIDINS220, IGSF21, and CACNA2D2 were incubated with: 1) rIgLON5-Fc protein, 2) irrelevant Fc protein (rCtrl-Fc; 110-HG-100, R&D Systems), 3) soluble IgLON5 at 2.5µg/ml, or 4) conditioned medium of untransfected HEK cells during 30 minutes. Cells were washed with PBS and fixed with 4% paraformaldehyde for 10 minutes. Binding of Fc-tagged recombinant proteins was determined by incubation with Alexa Fluor 594 goat anti-human Fcγ-fragment specific (109-586-098; Jackson Immunoresearch, Newmarket, UK). Binding of soluble IgLON5 was detected with a rabbit IgLON5 antibody (ab122763, Abcam) and anti-rabbit Alexa Fluor 594 secondary antibody (Thermo-Fisher Scientific) during 1 hour. Results were visualized in a confocal microscope ZEISS (LSM710, Carl Zeiss, Jena, Germany).

### 2.6 Effects of IgLON5-ab on IgLON5-protein interactions

rIgLON5-Fc and rCtrl-Fc proteins were biotinylated at 500 µg/mL with biotin-(long arm)-NHS following the datasheet (SP-1200, Vector Laboratories, Burlingame, CA, USA). Biotinylated rIgLON5-Fc (biot-rIgLON5-Fc) or biot-rCtrl-Fc protein were added to the media of cultures of hippocampal neurons or HEK cells transfected with the plasmids indicated above at 2.5 ug/mL for 30 min. To visualize the interactions, after washing, coverslips were fixed with 4% paraformaldehyde in PBS and incubated for 1 hour with Alexa Fluor 488-streptavidin (S11223, Thermo-Fisher) which specifically binds to biotinylated proteins.

To investigate if IgLON5-abs block the interaction of IgLON5 with its binding partners, parallel studies were performed in which biot-rIgLON5-Fc or biot-rCtrl-Fc were preincubated for 2 hours at 37°C with: 1) CSF diluted 1:2, or total purified IgG (2mg/mL) from 4 patients with the following IgLON5-IgG4 percentage of total IgLON5-IgG (patient #1 = 76%; #2 = 83%; #3 = 0%, and #4 = 100%); 2) NMDAR-IgG or 3) control IgG from blood donors (Ctrl-IgG) or CSF from a patient with Alzheimer’s disease.

## 3 Results

### 3.1 IgLON5 is released from the cell surface *via* ectodomain shedding

**Figure 1:**
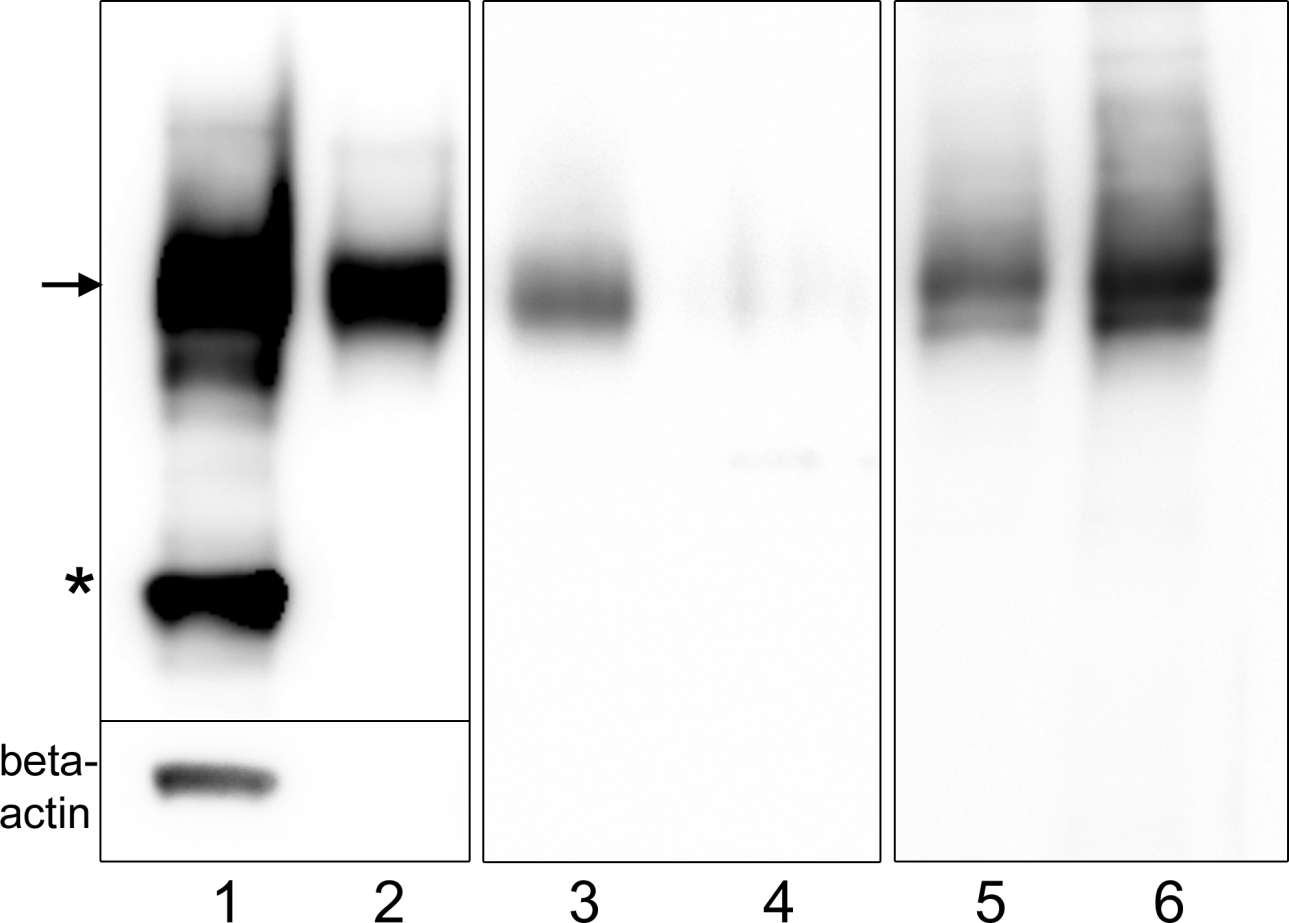
Immunoblots showing that IgLON5 is spontaneously shed and cleaved at the GPI-anchor. Immunoblots of different protein preparations from IgLON5-HEK probed with IgLON5 and actin antibodies: cell lysate (1); media obtained from 10 P60 plates with IgLON5-HEK cells (2); membrane preparations from one P60 plate untreated (3) and treated with PI-PLC (4); media from one P60 plate untreated (5) or treated with PI-PLC (6). Arrow indicates the glycosylated band of IgLON5 of approximately 60 kDa and the asterisk points the 37 kDa unglycosylated band. Only the lysate lane shows a beta-actin band, ruling out that the IgLON5 band detected in the other lanes is derived from dead IgLON5-HEK cells.

Immunoblot analysis showed that IgLON5 is present in the media of IgLON5-HEK cells indicating that is constitutively shed. When we treated IgLON5-HEK cells with the PI-PLC enzyme, which cleaves GPI-anchored proteins, we found that IgLON5 levels dramatically decreased from membrane preparations whereas IgLON5 in the culture media of PI-PLC-treated cells increased (**Figure 1**). Soluble IgLON5 had an approximate molecular weight of 60 kDa demonstrating that the protein shed is fully glycosylated and the cleavage site is close to the membrane, thus nearly a full-length fragment of the protein is released (**Figure 1**). Soluble IgLON5 was also found in the media of CGNs but in lower concentration (data not shown).

### 3.2 Interactome of IgLON5 and effect of IgLON5 antibodies on rat neurons

**Figure 2:**
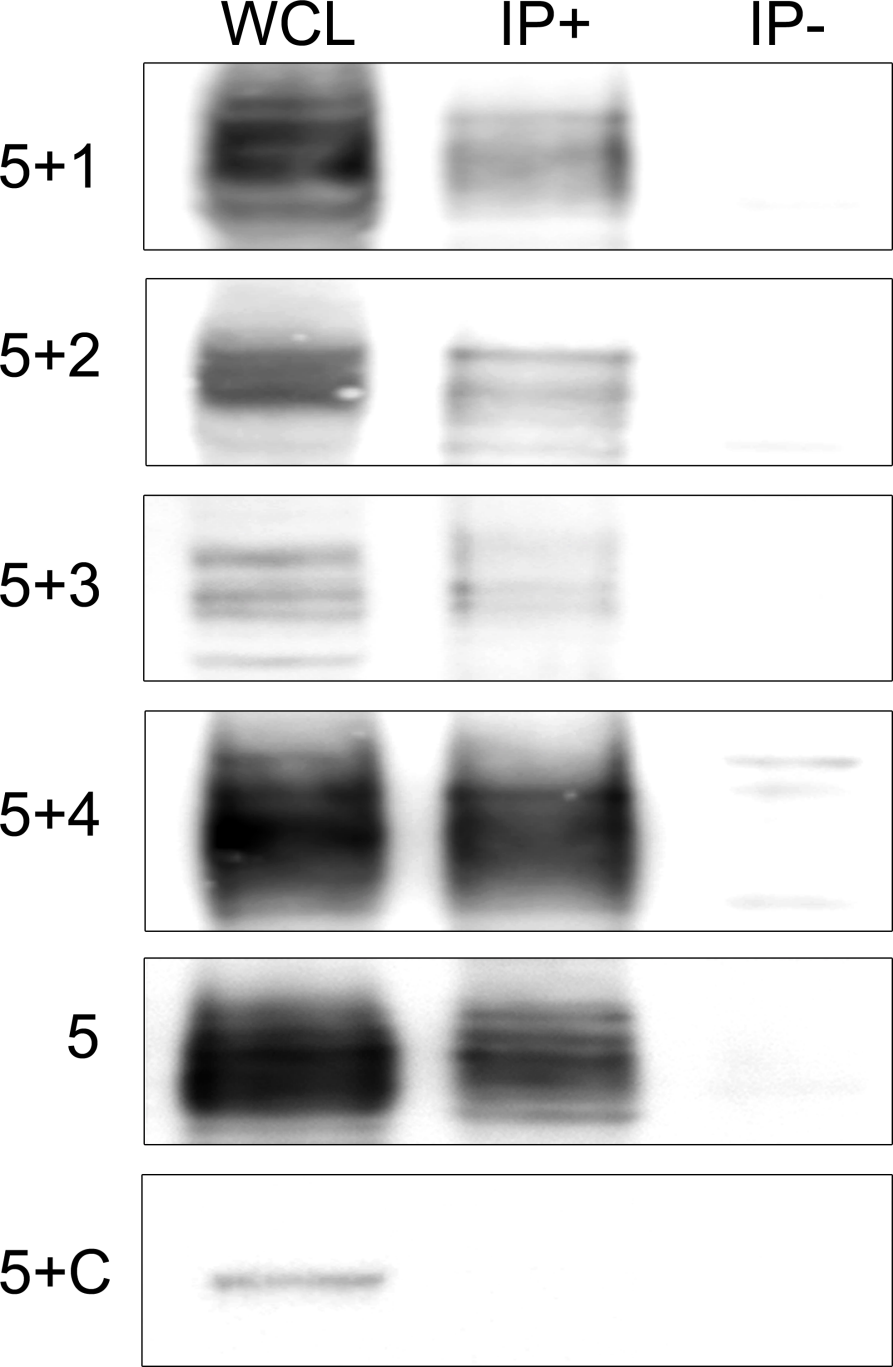
Immunoblots of co-tranfected HEK cells immunoprecipitated with patients’ IgLON5 antibodies. HEK cells co-transfected with IgLON5 and each of the other IgLON family members: first row (IgLON5 + IgLON1), 2nd row IgLON2 (5+2), 3rd row (5+3), and 4th row (5+4). The 5th row corresponds to HEK cells transfected only with IgLON5, and the 6th row HEK cells co-transfected with IgLON5 and CASPR2 (5+C). Left column: Whole cell lysates (WCL); middle column: Immunoprecipitates of transfected HEK cells using patients’ IgLON5-abs (IP+); right column: Immunoprecipitates of transfected cells using Ctrl-abs (IP). Each row was incubated with commercial antibodies against the corresponding proteins: IgLON1, 2,3,4,5 and CASPR2.

To gain insight into the physiological function of IgLON5 and to identify potential binding partners with transmembrane domain that could act as scaffolding proteins in the interaction of IgLON5 with the cytoskeleton, we investigated the IgLON5 interactome in CGN immunoprecipitated with IgLON5-abs. IgLON5 peptides were found in all the immunoprecipitations performed with IgLON5-abs and in none of the controls. In addition to other members of the IgLON family, cell membrane proteins that consistently co-precipitated with IgLON5 included two GPI-anchor proteins; immunoglobulin superfamily member 21 (IGSF21) and voltage-dependent calcium channel subunit alpha-2/delta-2 (CACN2D2A), and the transmembrane protein KIDINS220 (kinase D-interacting substrate of 220-kDa), also known as ARMS (ankyrin repeat-rich membrane spanning) (**Table 1**). These membrane proteins were specifically identified by mass-spectrometry as *bona-fide* interactors of IgLON5 and were selected for further analysis. To confirm *cis*-interactions, these proteins were co-transfected with IgLON5 in HEK cells and immunoprecipitated with IgLON5-abs. Immunoblots of the immunoprecipitates identified IgLON1 to 5 (**Figure 2**) but not any of the 3 additional proteins identified by mass-spectrometry (data not shown).

**Table 1:** Membrane proteins identified as *bona-fide* IgLON5 interactors.

Protein	Accession (NCBI)	Score	Coverage (%)	Peptides	IP*
IgLON5	XP_218634.5	277.6	38.69	11	5/5
IgLON3	NP_058938.1	160.4	23.67	7	4/5
IgLON2	XP_017451356.1	119.3	10.5	3	4/5
IgSF21	NP_001258383.1	62.9	6.41	3	2/5
IgLON1	XP_017450905.1	48.2	7.4	3	4/5
IgLON4	NP_067714.1	42.6	8.62	2	4/5
CACNA2D2	XP_006243799.1	34.3	0.87	1	3/5
KIDINS220	XP_017449472.1	28	0.47	1	2/5

* Number of immunoprecipitations where the protein is identified.

IgSF21: Immunoglobulin superfamily member 21 precursor; KIDINS220: Kinase D-interacting substrate of 220 kDa isoform X20; CACNA2D2: Voltage-dependent calcium channel subunit alpha-2/delta-2.

**Figure 3:**
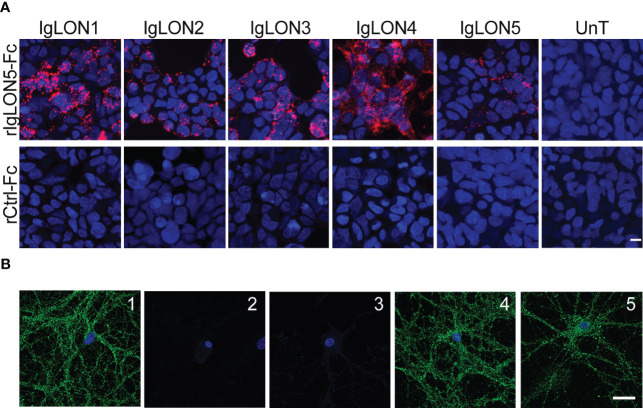
**(A)** Soluble IgLON5 is able to self-interact in trans and to bind to IgLON1-5. HEK cells, un-transfected (Unt) and transfected with IgLON1 to 5 incubated with rIgLON5-Fc protein (Top row) or rControl-Fc protein (second row). IgLON5 reactivity was detected with anti-Fc IgG-594 (Red fluorescence). The strongest IgLON5 interaction is observed with IgLON1, 3 and 4. No IgLON5 immunoreactivity was detected in untransfected HEK cells or when cells were incubated with rControl-Fc protein (bottom row). **(B)** Effect of IgLON5-abs on the binding of soluble IgLON5 to hippocampal neurons. IgLON5 immunofluorescence of live rat hippocampal neurons incubated with biotinylated rIgLON5-Fc (panel 1) or biotinylated rCtrl-Fc (2); Pre-incubation of biotinylated rIgLON5-Fc with CSF from patient 1 (100% IgLON5-IgG4) abolished the cell surface binding of IgLON5 (3), whereas pre-incubation with CSF from a patient with Alzheimer disease (4) or anti-NMDAR encephalitis (5) did not interfere with the binding of IgLON5 to the cell surface of neurons. Scale bar=20um.

To search for the presence of self-trans-interactions of IgLON5 or trans-interactions with the identified binding partners, we incubated soluble IgLON5 or rIgLON5-Fc with HEK cells transfected with IgLON1 to 5, KIDINS220, IGSF21 or CACNA2D2. HEK cells transfected with IgLON5 were probed with rIgLON5-Fc as Fc-tag allowed us to discriminate between the added IgLON5 and the IgLON5 attached to the membrane. We observed robust IgLON5 immunoreactivity after HEK cells transfected with IgLON1 to 5 were incubated with soluble IgLON5 but not after incubation with controls; media from untransfected HEK cells or Ctrl-Fc protein (**Figure 3A**). Neither soluble IgLON5 nor rIgLON5-Fc was able to bind to HEK cells transfected with the other identified binding partners (data not shown).

To demonstrate that soluble IgLON5 binds to the cell-surface of neurons, we incubated rIgLON5-Fc or Ctrl-Fc protein in the medium of cultures of live rat hippocampal neurons and found that rIgLON5-Fc but not Ctrl-Fc protein bound to the neuronal membrane. To further investigate a potential effect of IgLON5-ab on interactions between IgLON5 and other IgLONs, we pre-incubated purified IgG or CSF from 4 patients with different IgLON5-IgG4 subclass percentage (from 0 to 100%) with biot-rIgLON5-Fc or biot-rCtrl-Fc. Samples from the four patients completely blocked the binding of biot-rIgLON5-Fc to IgLON1-5 expressed in HEK cells (data not shown) and to hippocampal neurons independent of the IgG4 content. Neither antibodies from healthy donors nor antibodies against the NMDA receptor were able to block IgLON5 interactions confirming specificity (**Figure 3B**).

## 4 Discussion

Although substantial progress has been made in describing the immunological and clinical features of anti-IgLON5 disease (10, 11), the physiological function of IgLON5, the target of patients’ antibodies is largely unknown. Here we show that IgLON5 undergoes ectodomain shedding and interacts with the other members of the IgLON family, and IgLON5-abs disrupt this interaction. This effect represents a second potential pathogenic mechanism in addition to the irreversible internalization of IgLON5 clusters from the neuronal surface mediated by IgG1 IgLON5-abs that we previously described (5).

Our *in vitro* experiments in cultures of CGN and IgLON5-transfected HEK cells indicate that IgLON5 is spontaneously shed and that IgLON5 concentration in the media increases when cultures are exposed to PI-PLC enzyme that specifically cleaves GPI-anchored proteins. The molecular weight of the IgLON5 band that we detected in the media of untreated cells was similar to that observed after incubation with the PI-PLC enzyme indicating that in physiological conditions IgLON5 is cleaved at the level of the GPI anchor close to the membrane. These findings are in line with the previous demonstration that other IgLON family members also undergo ectodomain shedding and are cleaved from the cell membrane by matrix metalloproteinases (3). Ectodomain shedding of several neuronal cell adhesion proteins (i.e., NCAM or N-Cadherin) has been implicated in neurobiological mechanisms such as neuronal plasticity, axonal guidance, or cell migration (12). In the case of IgLONs, *in vitro* studies have shown that cleaved heterodimers of IgLON1 and 2 inhibit initiation of neurite outgrowth from chick neurons through the interaction with the IgLONs expressed in the neuronal membrane (13). Our study also shows that soluble IgLON5 can establish intercellular interactions. Additional studies will be necessary to ascertain the final effects of this interaction and if the effects are mediated by IgLON5 alone or through the formation of heterodimers with other IgLON family members.

IgLONs do not have a transmembrane domain and how they initiate downstream signal transduction pathways is presently unknown. The most plausible explanation is that IgLONs interact with other transmembrane receptors. For example, the GPI-anchored protein cell adhesion molecule transient axonal glycoprotein-1 (TAG-1), forms part of a protein complex with contactin-associated protein-like 2 (CASPR2) and shaker-type voltage-gated potassium channels (Kv1.1 and Kv1.2) which have domains that initiate downstream signaling pathways (14). However, the transmembrane receptors for IgLONs remain largely unknown. In an *in vitro* model, IgLON4 interacted with receptor tyrosine kinase fibroblast growth factor receptor 2 (FGFR2) to form a molecular complex that regulates extracellular signal-regulated kinase (ERK) and protein kinase B (AKT) pathways which control neuronal migration and maturation (15).

Our immunoprecipitation studies confirm that IgLON5-abs co-precipitate IgLON5 along with the other members of the IgLON family forming a protein complex. This result was expected considering previous studies demonstrating that homophilic and heterophilic interactions between the other IgLON family members are necessary for the effects of IgLONs on neurite outgrowth during development (16). In addition to other IgLON family members, our unbiased mass spectrometry approach identified three membrane proteins, IGSF21, CACNA2D2, and KIDINS220, as *bona-fide* interactors. KIDINS220 is of particular interest because this membrane protein modulates the development and maturation of axons and dendrites and regulates the activity of components of the actin and microtubule cytoskeleton (17) and we have previously shown that IgLON5-abs disrupt the cytoskeletal organization in cultured rat hippocampal neurons resulting in dystrophic neurites and axonal swelling (6). However, unlike the IgLONs, IgLON5-abs failed to co-precipitate these 3 proteins when co-transfected with IgLON5 in HEK cells. Possible explanations are a higher sensitivity of the mass spectrometry technique or that the interactions are transient or only detectable in the neuronal membrane.

The predominant subclass of IgLON5-abs is IgG4 but the proportion of IgG1 and IgG4 IgLON5-abs varies among patients (5). We previously demonstrated that IgLON5-abs of the IgG1 subclass produce an irreversible decrease of IgLON5 clusters on the cell surface by cross-linking and internalization (5). Here we show another potential pathogenic mechanism in which patients’ IgLON5-abs disrupt the interaction of the secreted IgLON5 ectodomain with its binding partners in the neuronal membrane. Disruption of protein-protein interactions is characteristic of IgG4-abs (18), however, we found that this phenomenon was not exclusively dependent of IgG4 IgLON5-abs as sera with only IgG1 antibodies produced the same effect. Our findings are in line with pathogenic mechanisms described for other autoimmune encephalitides associated with neuronal surface antibodies. For example, Leucine-rich glioma-inactivated 1 antibodies (LGI1-abs) block the interaction of soluble LGI1 to its binding partners ADAM22 and ADAM23 (19). Similarly, CASPR2-abs inhibit the interaction of CASPR2 with its ligand TAG1 resulting in a reduction of neuronal surface CASPR2 clusters without affecting TAG1, suggesting selective CASPR2 internalization (20). Neither model evaluated if the effects were mainly mediated by IgG1 or IgG4 antibodies.

Our results indicate that the physiological ectodomain shedding of IgLON5 and its interaction with the other members of the IgLON family likely mediate the effects of IgLON5 as has been demonstrated for other IgLONs. The ability of patients’ IgLON5-abs to interfere with these interactions represents another potential pathogenic mechanism in addition to the antibody-mediated irreversible internalization of IgLON5 (5).

## Data availability statement

The data presented in the study about immunoprecipitation results are deposited in the dryad repository accession number: https://datadryad.org/stash/share/YX-zapxd15SZntbA3WSK7LpC0SSAIhKoosRcGE_ex2M.

## Author contributions

1) Conception and design of the study LS, JL, FG, JD. 2) Acquisition and analysis of data LS, JL, CG, JS, FG, ABS, AS, IK, RH. 3) Drafting a significant portion of the manuscript or figures LS, FG, JL, JD. All authors contributed to the article and approved the submitted version.
