# Corrigendum: Patients’ IgLON5 autoantibodies interfere with IgLON5–protein interactions

**DOI:** 10.3389/fimmu.2023.1233632

**Published:** 2023-06-29

**Authors:** Jon Landa, Ana Beatriz Serafim, Carles Gaig, Albert Saiz, Inga Koneczny, Romana Hoftberger, Joan Santamaria, Josep Dalmau, Francesc Graus, Lidia Sabater

**Affiliations:** ^1^ Neuroimmunology Program, Fundació de Recerca Clínic Barcelona-Institut d’Investigacions Biomèdiques August Pi i Sunyer, Universitat de Barcelona, Barcelona, Spain; ^2^ Service of Neurology, Hospital Clinic de Barcelona, Barcelona, Spain; ^3^ Division of Neuropathology and Neurochemistry, Department of Neurology, Medical University of Vienna, Vienna, Austria; ^4^ Comprehensive Center for Clinical Neurosciences and Mental Health, Medical University of Vienna, Vienna, Austria; ^5^ Department of Neurology, University of Pennsylvania, Philadelphia, PA, United States; ^6^ Institució Catalana de Recerca i Estudis Avançats (ICREA), Barcelona, Spain

**Keywords:** anti-IgLON5 antibody encephalopathy, anti-IgLON5 disease, IgG4, interactome, neurons, IgLON5 antibodies

In the published article, there was an error in the Funding statement. We did not include that Ana Beatriz Serafim is a recipient of a Doctoral Fellowship grant from Fundação para a Ciência e Tecnologia (FCT) contract number 2022.13121.BD and should be acknowledged. The correct Funding statement appears below.

